# Low Neonatal Mortality and High Incidence of Infectious Diseases in a Vietnamese Province Hospital

**DOI:** 10.1155/2016/2087042

**Published:** 2016-08-11

**Authors:** Binh T. T. Ho, Alexandra Y. Kruse, Hue T. H. Le, Phuong N. Cam, Freddy K. Pedersen

**Affiliations:** ^1^Neonatal Intensive Care Unit, Children's Hospital 1, Ho Chi Minh City, Vietnam; ^2^Paediatric and Adolescence Clinic, Juliane Marie Centre, Rigshospitalet, Copenhagen, Denmark; ^3^Paediatric Intensive Care Unit, Dong Thap General Hospital, Dong Thap Province, Vietnam

## Abstract

*Background*. Neonatal deaths constitute the majority of child mortality in Vietnam, but studies are scarce and focus on community settings.* Methods*. During a 12-month period, all sick neonates admitted to a pediatric department in a province hospital were studied. Potential risk factors of death covering sociodemographic factors, pregnancy history, previous neonatal period, and status on admission were registered. The neonates were followed up until discharge or death or until 28 completed days of age if still hospitalized or until withdrawal of life support. The main outcome was neonatal death.* Results*. The neonatal mortality was 4.6% (50/1094). In a multivariate analysis, four associated risk factors of death were extremely low birth weight (OR = 22.9 (2.3–233.4)), no cry at birth (OR = 3.5 (1.3–9.4)), and cyanosis (OR = 3.3 (1.2–8.7)) and shock (OR = 12.3 (2.5–61.5)) on admission. The major discharge diagnoses were infection, prematurity, congenital malformations, and asphyxia in 88.5% (936/1058), 21.3% (225/1058), 5.0% (53/1058), and 4.6% (49/1058), respectively. In 36, a discharge diagnosis was not registered.* Conclusion*. Infection was the main cause of neonatal morbidity. Asphyxia and congenital malformations were diagnosed less frequently. The neonatal mortality was 4.6%. No sociodemographic factors were associated with death. Extreme low birth weight, no cry at birth, and cyanosis or shock at admission were associated with death.

## 1. Introduction

Globally, The Millennium Development Goal to reduce child mortality by two-thirds was not achieved yet [1–10]. A recent Lancet paper estimated that about 44 percent of all under-5 deaths occur in the neonatal period [11]. In recent years, neonatal mortality has been a priority on the global health agenda [12]. Studies on neonatal mortality in low-middle income countries have increased but are still scarce compared to the magnitude of the problem and most focus on community settings [3–5, 7–10, 13, 14].

In accordance with the international trend, child mortality has decreased significantly in Vietnam within the recent years (22/1000 live births in 2011), but reduction in neonatal mortality is lagging behind with a reported Neonatal Mortality Rate (NMR) of 12/1000 live births [9, 12, 14–22]. Among the reported deaths, the majority (63–78%) were related to prematurity, infection, and asphyxia in accordance with the global estimates [1–3, 5, 8, 11, 13, 23]. Most neonatal deaths are expected to occur in a health care facility, since this is where 79% deliver, and the first days after delivery are known to carry the highest risk of death [18, 19]. However, peer reviewed studies on neonatal hospital mortality in Vietnam are few [5, 9, 14].

Aim of the study was to register the number of neonatal deaths, risk factors for death at admission, and causes of neonatal morbidity in a general province hospital.

## 2. Materials and Methods

### 2.1. Study Setting

Dong Thap Province in South Vietnam on the border to Cambodia has a population of 1.8 million with 31200 live births annually and NMR of 17/1000 live births [24]. The study was conducted in the main hospital of the province, Dong Thap Province General Hospital. It is a secondary level hospital with 700 beds, with pediatric and obstetric departments and 6500 deliveries annually [25]. The pediatric department includes a pediatric ward and a pediatric intensive care unit. Neonates are admitted to the pediatric department internally from the obstetric ward and externally from home or referred from lower levels of care in the province [25]. Nasal continuous positive airway pressure, mechanical ventilation, and parenteral nutrition are part of the neonatal care offered in the hospital. Dong Thap Hospital is comparable to the other 12 main provincial hospitals in Southwestern Vietnam with similar socioeconomic conditions and health system structure. The Children's Hospital 1 (CH1) in Ho Chi Minh City provides the most specialized neonatal care in southern Vietnam and is the referral hospital for Dong Thap Hospital and other southern provincial hospitals.

### 2.2. Study Design and Material

We conducted a prospective study including all sick neonates admitted to the pediatric department of Dong Thap Hospital during a 12-month period from 1/4/2010 to 31/3/2011. The participants were followed up until discharge (to home or referral to a tertiary level hospital) or death or until 28 completed days if still hospitalized or until withdrawal of life support (WLS). The following groups were excluded: healthy neonates admitted to the obstetric ward with their mothers for standard postnatal care, readmissions during the neonatal period, and lack of family consent to participate in the study.

### 2.3. Data Collection

At admission, the doctor receiving the neonate completed a structured questionnaire together with the family. Data collected covered sociodemographic factors, pregnancy history, previous neonatal period, and admission status (see Appendices  I and II in the Supplementary Material available online at http://dx.doi.org/10.1155/2016/2087042). At the end of the follow-up period, data on outcome and ICD-10 diagnoses (one or more diagnoses were assigned) were collected from the hospital computer registry [18, 26]. Data on death, WLS, and referral in severe condition were also registered in the ward book by the nurses and in a separate list by the head of the pediatric intensive care unit. WLS cases were followed up until 28 completed days of age by telephone. Birth weight was recorded on all deliveries in the hospital during the study period.

### 2.4. Outcome

The outcome was defined as death or WLS death in the neonatal period (≤28 days). WLS was defined as withholding/withdrawing of manual or mechanical ventilation and discharge alive to die at home [27, 28].

Morbidity data were based on the registered discharge diagnosis.

### 2.5. Data Analysis

Data were entered in Microsoft Access 97 and analyzed in SPSS. Ten percent of the data were entered in duplicate. When compared, the discrepancies between entries were less than 5%.

Associations between risk factors and outcome were analyzed in multivariate logistic regression using backwards elimination if *p* > 0.20. Unadjusted and adjusted odds ratios (OR) and 95% confidence intervals (CI) are reported.

### 2.6. Ethics

The ethical board of the study hospital approved the study. Oral informed consent was sought from the families of the neonates participating. If they rejected or were not present, only basic data were registered from the standard registration. For neonates who had WLS, consent was obligatory prior to follow-up by telephone.

## 3. Results

A total of 1171 sick neonates were admitted to the pediatric department during the 12-month study period. Ten cases rehospitalized in the neonatal period were excluded. Data were missing on 67 cases (5.8%), leaving 1094 cases available for analysis. No families declined participation. There were no deaths or WLS in the missing group or the excluded group.

**Figure 1:**
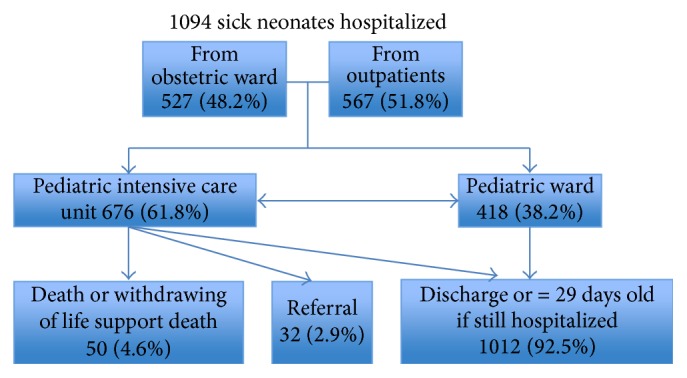
Flow chart of the study population.

As shown in Figure 1, about half (48.2% or 527/1094) were admitted from obstetrics ward and half from outside. Of the latter 37.7% or 412/1094 were admitted from home and 13.9% or 152/1094 were referred from other health care facilities.

During the study period, a total of 6057 live birth deliveries were performed in the hospital, (i.e., approximately a fifth of all live births in Dong Thap Province). Of these 919 (15.2%) were of low birth weight (LBW < 2500 g) and 59 (1.0%) were of very low birth weight (VLBW < 1500 g) (Appendix  III). There were 527 (8.7%) of the babies, delivered in the hospital, that were transferred to pediatric care.

Table 1 shows the characteristics of the study population including male-to-female ratio, gestational age, birth weight, and age of admission in 2 groups from obstetric ward and from outpatients.

The male-to-female ratio in the study was 1.24. The proportion of premature (<37 weeks) and very premature babies (<32 weeks) was 24.8% and 6.9%; almost a third (30.4%) were of LBW and 5.1% were of VLBW.

Almost a third (29.9% or 327/1094) were admitted in the first day of life: 48.6% (256/527) from the obstetric ward and 12.5% (71/567) from outpatients.

17 cases (1.6%) were ethnic minorities and 76.5% or 13/17 of them were admitted from outside. 906 cases of the 1094 (82.8%) had attended recommended antenatal care including a minimum of 3 consultations. Approximately half (47.6% or 521/1094) had a normal delivery, 25.3% (277/1094) had a cesarean delivery, and only 0.8% (9/1094) were delivered at home. 64.6% (707/1094) were breastfed with or without formula milk.

Of the 1094 neonates studied, 654 (59.8%) were not discharged after birth and 669 (61.2%) had symptoms for less than 1 day before admission. The most frequent danger symptoms before admission according to parents or caretakers were symptoms related to feeding, breathing, and yellow skin: 11.2% (123/1094), 17.4% (190/1094), and 27.1% (297/1094), respectively. Cyanosis, lethargy/comatose condition, and paleness were detected at admission by the doctor in 10.3% (113/1094), 5.4% (59/1094), and 4.1% (45/1094); symptoms observed by the caretaker like blue skin, movement only on stimulation, difficulty in waking up, or pale skin were less recognized: 5.1% (56/1094), 0.5% (6/1094), 0.3% (3/1094), and 0.2% (2/1094), respectively.

Of the 567 external admissions, 136 (24.0%) were transported by ambulance from a health care facility and 39 (28.7%) of 136 deteriorated during transportation.

At admission, 5.9% (64/1094) of the neonates were in severe condition with severe respiratory failure (2.0%), shock (2.4%), or coma/lethargy (5.5%).

Table 2 shows the diagnoses assigned at follow-up in 1058 of 1094 (in 36 a discharge diagnosis was not registered) and compares figures for the same diagnosis at Children's Hospital 1, Ho Chi Minh City. Infection was assigned in 88.5% of the 1058 neonates in the study hospital, and Table 3 shows some characteristics of the neonates with infection.  Table 4 shows the multivariate analysis of risk factors of neonatal hospital death.

### 3.1. Risk Factors of Hospital Death

Neonatal death was registered in 50 neonates, 2 cases of which were WLS and died in the neonatal period, corresponding to a mortality rate of 4.6% (50/1094). Another 3 WLS cases were alive beyond 28 completed days of age. Further 32 severely ill cases were transferred to tertiary level in Ho Chi Minh City out of which 2 died, 21 cases were alive throughout the neonatal period, and 9 cases were lost to follow-up.

The multivariable logistic regression found extremely low birth weight (OR = 22.9 (2.3–233.4)), no cry at birth (OR = 3.5 (1.3–9.4)), and cyanosis (OR = 3.3 (1.2–8.7)) and shock at admission (OR = 12.3 (2.5–61.5)) associated with increased risk of death.

## 4. Discussion

This prospective descriptive study included 94.2% of all admitted neonates to Dong Thap Province hospital during the 12-month study period with no missing cases of death or WLS. It may therefore be considered representative for the situation in provincial hospitals in at least the southwestern part of Vietnam where socioeconomic conditions, cultural traditions, delivery traditions, referral practices, and levels of care are comparable.

Among the 1094 neonates included, the mortality was 4.6%. This figure does not include 2 cases (0.2%) that died during the neonatal period after referral to Children's Hospital 1, Ho Chi Minh City, because these deaths may be influenced by factors other than those of the provincial hospital. Nine referred cases were not available for follow-up, which also adds some uncertainty to the total mortality of the whole cohort. If all cases unavailable for follow-up after referral were assumed dead and those two cases known to be dead after referral were added, the total mortality in the full cohort would be 5.6% (61/1094). The study only registered events during hospitalization within the neonatal period and did not follow up cases hospitalized beyond 28 days of age, where some deaths could have occurred, as could have been the case in the 3 WLS patients that were alive at 28 days.

To our knowledge, no data of neonatal mortality in hospital in Vietnam with the exception of that from the tertiary Children's Hospital 1 from our group has been published [5]. In a systematic review of the burden of neonatal mortality and morbidity in the ASEAN region published in 2012, among 20 publications included, there were only three studies from Vietnam all focusing on community neonatal mortality [29].

The mortality rate of 4.6% was low, especially compared to situations where advanced treatment is not available [1, 2, 16, 23, 30–32]. Hospital mortality rates of 13.8% have been reported from other low and middle income countries [16, 23]. Many, however, were admitted to hospital due to simple jaundice or mild respiratory problems that could have been admitted to lower level health care facilities. Another likely explanation of the low mortality in this study is the low proportion of VLBW (5.1%) and asphyxiated (4.6%) and congenital malformations (5.0%) in the study population, since all are associated with a high neonatal mortality globally [1–3, 14, 23, 31]. The causes of death in this material are being investigated in detail in another study using a death audit procedure.

Babies born very preterm (gestational age < 32 weeks) and/or with very low birth weight (birth weight < 1500 g) had mortality rates of over 50% in many low-resource settings of Asia or Africa [8, 12, 16, 33] and are at a higher risk for long-term disabilities and impairments [8, 12, 16]. In low-middle income countries, most early neonatal deaths are caused by prematurity [8, 12, 16, 34]. Gestational age is an important factor of neonatal mortality, but the relation to hospital death in our study was not statistically significant in the multivariate analysis. However, extremely low birth weight was the strongest risk factor of death (OR adjusted 22.9 (2.3–233.4)). “No cry at birth” with OR adjusted = 3.5 (1.3–9.4) is a symptom that reflects maladaptation of the neonate in the out-of-uterus environment and thus may also be due to severe prematurity, asphyxia, or congenital malformations.

Shock at admission was a strong predictor of death. Indeed, treatment of shock in the neonate was a challenge in Dong Thap Hospital, where there were no treatment guidelines and lack of possibilities for monitoring. Severe respiratory failure disappeared in the multivariate analysis, and cyanosis at admission remained one of four final risks factors.

No sociodemographic factors were associated with hospital death. It was an important finding of the study in Dong Thap Hospital and Children's Hospital 1, Ho Chi Minh City, since these factors have been shown to be of importance for infant survival in other low and middle income countries [1, 2, 4, 5, 12, 16, 21, 34, 35]. In our total study, the male-to-female ratio was 1.24, in patients from outside was 1.17, and in those from obstetric ward was 1.32; this probably reflects the predominance of males in the community (1.09–1.11) as well as possibly male infant vulnerability [24, 36]. The ethnic minority rate was 1.6%, higher than the rate in the Dong Thap population (0.6%) [24], probably reflecting a different health seeking behavior of the ethnic minority group in southern Vietnam. Neither gender nor ethnic minority, however, was related to hospital death. These findings in our study showed no inequity in access to care in treatment and neonatal outcome in Vietnam due to the level of the parent's education, ethnic origin, or the gender of the neonate. We found that young neonates admitted from the obstetric ward were more than those from outside; this probably reflects that women with higher risk of complication of pregnancy or delivery more often are admitted to the province hospital. Besides that, we have not recognized any selection bias for referral. Referral to tertiary hospital is done when highly specialized treatment is needed, for example, neonatal surgery.

The discharge diagnosis of asphyxia was similarly low in Dong Thap Hospital and Children's Hospital 1, Ho Chi Minh City [5], but the limited data available in Vietnam and the ASEAN region probably do not reveal the real burden of birth asphyxia in this region [14]. A study of incidence of hypoxic ischemic encephalopathy in Dong Thap Hospital and a tertiary obstetrics hospital in Ho Chi Minh City, however, is ongoing.

The rate of congenital malformations was 5.0%, lower than that in Children's Hospital 1, Ho Chi Minh City (15.0%) [5]. This difference could be explained by the limited capacity of prenatal and postnatal malformation diagnosis and lack of neonatal surgery in Dong Thap Hospital, whereas Children's Hospital 1, Ho Chi Minh City, is a tertiary hospital and a center of neonatal surgery in South Vietnam.

Infection was the main diagnosis assigned to 88.5% of the patients. Other studies from Vietnam and from other low-middle income countries have also shown high rates of 38.0% to 62.8% [2, 5, 16, 23]. Unspecified infection was assigned to 35.6% as there were no specific diagnostic criteria for assigning the diagnosis by the treating physician. It is therefore likely to be based on a clinical impression, which may be incorrect. But even if the cases classified as unspecific infections are deducted, an infection rate of the resulting 52.9% is high. According to global figures, infections cause 30–50% of the neonatal mortality burden in low income countries [8, 23], and the incidence of late-onset sepsis was higher in Asia than in resource-rich countries [37]. This high figure may reflect insufficient hygienic procedures at home or in the hospital [23, 29]. The rate of infection was highest in the group transferred from the other health care facilities (96.1% or 146/152), followed by those from the obstetrics ward (85.0%, 448/527) and 83.0% or 342/412 from home. This suggests that nosocomial infection in hospitals in the region may be a problem. An infection diagnosis is likely to affect antibiotic prescription and thus favor a high prevalence of bacteria resistant to antibiotics [14, 23, 29]. To prevent the growing problem of antibiotic resistance, specification of diagnostic infection criteria will be important. Further it may contribute to improving efforts to identify other relevant diagnoses and hence proper management of the neonatal cases. Health education of mothers and training of hospital staff on proper hygienic measures and infection control are likewise important to reduce the incidence of neonatal infection.

After finalization of the research, the training of medical staffs in basic newborn care was established and maintained. The neonatal infection diagnosis criteria and shock guidelines were updated. The infection control program in the hospital received more interest and the health education program for mothers and family members of sick neonates was realized within the scope of hospital.

## 5. Conclusions

This study of mortality and morbidity among 1094 consecutively admitted sick neonates in a general province hospital in South Vietnam contributes to the limited data on hospital admitted neonates in Vietnam. Infection was the main cause of neonatal morbidity. Asphyxia and congenital malformations were diagnosed less frequently than expected.The neonatal hospital mortality was 4.6%. No sociodemographic factors were associated with neonatal death. The risk factors statistically significantly related to death were extremely low birth weight, no cry at birth, and cyanosis or shock at admission. Introduction of specific diagnostic criteria for infection to reduce improper antibiotic use and the development of antibiotic resistance and health education of mothers and training of hospital staff in hygienic measures and infection control may help reduce neonatal infections. The instruction of updated shock guidelines is crucial.

## Supplementary Material

The supplementary material included 4 appendixes. Appendix I shows the registered data form for all sick neonates admitted to Dong Thap hospital. Appendix II shows all potential risk factors registered in the study. Appendix III shows the distribution of birth weight of all deliveries in obstetric department in Dong Thap hospital in the study period. Appendix IV shows the description of all potential risk factors in the study.

## Figures and Tables

**Table 1:** Characteristics of the study population.

	Obstetric ward (%) *N* = 527	Outpatient (%) *N* = 567	Total (%) *N* = 1094
Male/female	300/227 = 1.32	306/261 = 1.17	606/488 = 1.24
Gestational age			
<28 weeks	12 (2.3)	4 (0.7)	16 (1.5)
28–31 weeks	31 (5.9)	28 (4.9)	59 (5.4)
32–36 weeks	124 (23.5)	72 (12.7)	196 (17.9)
≥37 weeks	354 (67.2)	417 (73.5)	771 (70.5)
Birth weight			
≤1000 g	12 (2.3)	3 (0.5)	15 (1.4)
<1500 g	27 (5.1)	14 (2.5)	41 (3.7)
<2500 g	158 (30.0)	119 (21.0)	277 (25.3)
≥2500 g	330 (62.6)	425 (75.0)	755 (69.0)
Age of admission			
Day 1	256 (48.6)	71 (12.5)	327 (29.9)
Day 2–day 7	261 (49.5)	115 (20.3)	376 (34.4)
Day 8–day 28	4 (0.8)	365 (64.4)	369 (33.7)

Data on birth weight, gestational age, and age of admission were missed in 6, 52, and 22 cases, respectively.

**Table 2:** Distribution of diagnoses at discharge in Dong Thap Hospital.

Discharge diagnosis^1^	Dong Thap Hospital *N* = 1058^2^		Children's Hospital 1, Ho Chi Minh City *N* = 5763 [5]	
	Cases	%	Cases	%
Prematurity	225	21.3	385	6.7
Infection	936	88.5	3618	62.8
*Sepsis/meningitis*	*172*	*16.3*		
*Skin/umbilical infection*	*232*	*21.9*		
*Pneumonia*	*155*	*14.7*		
*Unspecified infection*	*377*	*35.6*		
Congenital malformations	53	5.0	864	15.0
Asphyxia	49	4.6	120	2.1
Jaundice	280	26.5	1060	18.4

^1^Each neonate had one or more diagnoses assigned.

^2^36 of 1094 did not have a discharge diagnosis registered.

**Table 3:** Characteristics of neonates with infection.

	Infection group *N* = 936	Total *N* = 1094	Percentage %
Admitted from			
Home	342	412	83.0
Other HCF^1^	146	152	96.1
Obstetrics ward	448	527	85.0
Birth weight			
≤1000 g	12	15	80.0
1001–1499 g	37	41	90.2
1500–2499 g	259	277	93.5
≥2500 g	628	755	83.2
Age of admission			
Day 1	302	327	92.4
Day 2–day 7	327	376	87.0
Day 8–day 28	307	369	83.2

^1^HCF: health care facility.

**Table 4:** Risk factors of neonatal hospital death.

Characteristics	OR adjusted	*p* value
*Birth weight*		
*≤1000 g*	22.9 (2.3–233.4)	*0.008*
1001–1499 g	4.9 (0.9–29.6)	0.087
1500–2499 g	2.8 (0.8–10.00)	0.114
*No cry at birth*	3.5 (1.3–9.4)	*0.012*
*Skin color at admission*		
Yellow	0.1 (0.0–1.7)	0.119
*Cyanosis*	3.3 (1.2–8.7)	*0.019*
Pale	2.0 (0.5–9.1)	0.364
*Shock*	12.3 (2.5–61.5)	*0.002*
