# Supplementary material for: Low Neonatal Mortality and High Incidence of Infectious Diseases in a Vietnamese Province Hospital
Source: Biomed Res Int. 2016 Aug 11;2016:2087042. doi: 10.1155/2016/2087042 (PMC4997011; doi:10.1155/2016/2087042)
Supplement: Supplementary file 1 — The supplementary material included 4 appendixes. Appendix I shows the registered data form for all sick neonates admitted to Dong Thap hospital. Appendix II shows all potential risk factors registered in the study. Appendix III shows the distribution of birth weight of all deliveries in obstetric department in Dong Thap hospital in the study period. Appendix IV shows the description of all potential risk factors in the study. [file 2087042.f1.pdf]

APPENDIX I: NEONATAL ADMISSION DATA REGISTRATION

To be completed for all infants ***<29 days*** *of age at admission*

A) Informed consent by family Y/N/not asked Date ___/___ / 200_

B) Basic data

1) Completed by doctor: ______________________________, OBS/NICU/PED
2) Mothers name: ____
3) Filed out with: mother/other/none/orphan Contact (name/tel): _______________
4) Admission day: __/___ / 200_ and hour: _______ (administration time)
5) Birth date: ___/___ / 200_ and hour: _____
6) Birth weight: _______ (g) Gender: M/F GA: ___ (weeks) Term/preterm/post term

C) Neonatal period *(prior to present admission* to DTH)

1) Feeding: Breast milk/formula/not started Breast feeding/bottle/NG-tube
2) If birth weight < 2000 g; kangaroo-mother-care performed: Y/N
3) Vaccinations: BCG Y/N Engerix Y/N
4) Duration of symptoms _______ (days from first symptom, according to family or referral letter)
5) Specification of symptoms (more options can be tagged):
   Floppy/difficult to wake up/movement only when stimulated/fever/difficulties breathing/difficulties eating/convulsions/skin colour yellow or blue or pale/ cord surroundings wet or red/abnormal stool/distended belly/ other: _________
6) Discharged after delivery: Y/N
7) Seen at HCF prior to present admission: Y/N *If Y*; No of contacts: __; for each specify:
   Level OPD/adm Date(s) Diagnoses Treatment

____

____

D) DTH reception *(present admission)*

1) Admitted from: home/OBS/HCF *If HCF*; Referral report: Y/N HCF Name: ________
   Level: Health centre/district hospital/province hospital/private hospital
2) *If from home/HCF;* Transport; Duration: __ (h) Distance: __ (km)
Mode: walking/motor/car/amb
   Only if ambulance, fill out; Accompanied by: family/nurse/doctor/none
   Evaluation by: accompanying staff/family/DTH;
   Equipment in use: incubator/air/oxygen/bag ventilation/ET tube/NG tube/suction/iv/infusion pump/monitor (spO2/HR)/none/others: _____________________
   Equipment failure/incorrect use/displace: Y/N If Y; Specify: ________________
   Deterioration of patient: Y/N If Y; Specify: ______________________________
   Stabilisation attempt: Y/N If Y; Specify: ______________________________
3) Admission (from home/other HCF/obstetrics) to: NICU/PED
4) Evaluation at admission (by first doctor receiving the patient), time: _________
   Weight: ____ (g) T ^{0} __ RR: ___ HR: ___ Sp02: ___/Fi02____
   Pink/blue/pale/jaundice Irritable Y/N Alert/lethargy/coma Grunting: Y/N
   Chest indrawing: no/moderate/severe Fontanel: normal/bulging/sunken
   Pulse: normal/weak CRT: normal/prolonged Other signs: ___________________
   Diagnoses: ____

Treatment: Respiratory support Y/N, If Y oxy/CPAP/ENT/bagging Vitamin K Y/N
Circulation support Y/N, If Y: volume/pressor/chest compression
Antibiotics: Y/N Other: ____

E) Demography

1) Mother's age: _______ (year of birth)
2) Mother's education (completed): ≤5 gr/6-12 gr/> 12 gr
3) Father's education (completed): ≤5 gr/6-12 gr/> 12 gr
4) Both parents and newborn living together: Y/N
5) Current address (according to family): village/town/city Province: __________________
6) Ethnic minority (father/mother non Kinh): Y/N If Y, specify: _______________________

F) Mother

1) Weight (prior to pregnancy): _______ (kg)
2) Chronic diseases: Y/N If Y; specify ____
3) *Other* pregnancies; abortions (spontaneous): ___ (no) Stillbirths: ___ (no)
Livebirths: __ Dead (after birth): Y/N If Y; age and cause: _____________________

G) Pregnancy

1) ANC visits: <3/≥ 3; trimester I ___ / II ___ / III ___ (no)
Tetanus booster: 1 / 2 / no Iron supplement: Y/N Ultrasound: Y/N If Y;
Abnormal findings: Y/N If Y; specify ____________________________________
2) Illness/complication: Y/N If Y: fever/UTI/vaginal bleeding/pre-eclampsia/others
If others, specify: ____
3) Treatment (during pregnancy, besides iron): Y/N If Y; specify: _______________________

H) Delivery

1) Birth place: home/public HCF/private HCF
If public HCF: health centre/district hospital/province hospital/regional hospital
2) Birth assistance: No professional assistance/TBA/midwife/doctor
3) Normal delivery: Y/N (according to family and file) *Only if N, fill out;*
Prolonged labour (>18h): Y/N
Meconium stained amniotic fluid: Y/N
Premature membrane rupture (>18h): Y/N If Y; antibiotics Y/N
Premature labour: Y/N If Y corticosteroid: Y/N
Breech: Y/N
Multiple: Y/N
Delivery mode: normal/vacuum extraction/forceps/caesarean section
If Caesarean; Planned/acute +/% contractions Indication: ______________
4) Resuscitation (immediately after delivery): Y/N, if Y;
Bag-ventilation: Y/N Intubation: Y/N Chest compression: Y/N
5) Cry after birth Y/N Apgar score: __ /1 min, __ /5 min, __/ __ min, __/__min
6) Vitamin K (im): Y/N Other treatment (at delivery) Y/N
If Y, specify: ____
7) Maternal complication: Y/N If Y; Abnormal bleeding intra/post partum/others
If others; Specify: ____________________________ Treatment: ____________________

APPENDIX II: POTENTIAL RISK FACTORS REGISTERED IN THE STUDY

**1.Socio-demographic**	**2.Pregnancy-delivery**	**3.Neonatal period**
Ethnic minority	Number of antenatal care visits	Discharge after birth
Mother's education	Delivery place	Admitted from
Father's education	Twin pregnancy	Symptom duration
Number of siblings	Normal delivery	Type of feeding
**4.Admission status**	Mode of delivery	Difficulties eating
Ambulance transport	Cry after birth	Difficult to wake up
Admission ward	Resuscitation	Movement only when stimulated
Age at admission	Vitamin K	Convulsions
Skin color	BCG	Difficulties breathing
Temperature	Engerix B	Skin color change
Severe respiratory failure	Gender	Abnormal stool
Shock	Birth weight	
CNS trouble	Gestational age	

APPENDIX III: DELIVERIES IN OBSTETRIC DEPARTMENT OF DONG THAP HOSPITAL

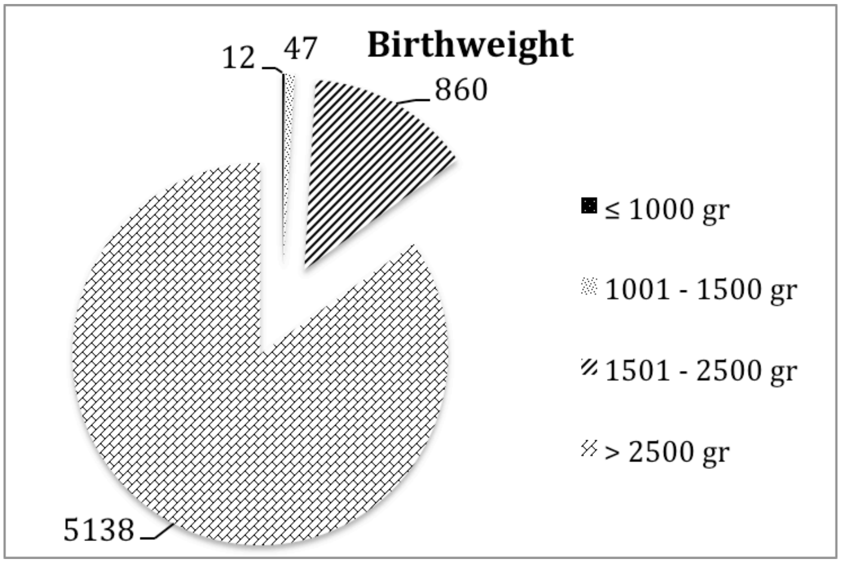

During the study period, the Obstetrics department had 6057 live births, approximately a quarter of total live births in Dong Thap province: 15.2% LBW and 1% VLBW.

APPENDIX IV: DESCRIPTION OF POTENTIAL RISK FACTORS

Characteristics of socio - demographics		No of cases (%)
Ethnic minority	Yes	17 (1.6)
	No	1055 (96.4)
	UK / miss	22 (2.0)
Mother's education	≤ class 5	293 (26.8)
	Class 6 – 12	661 (60.4)
	> Class 12	115 (10.5)
	UK / miss	25 (2.3)
Father's education	≤ Class 5	255 (23.3)
	Class 6 – 12	671 (61.3)
	> Class 12	142 (13.0)
	UK / miss	26 (2.4)
Number of siblings	0	630 (57.7)
	1	332 (30.3)
	2 – 4	92 (8.4)
	UK / miss	40 (3.6)

Characteristics of pregnancy- delivery		No of cases (%)
Number of antenatal care visits	< 3	145 (13.3)
	≥ 3	906 (82.8)
	UK / miss	43 (3.9)
Delivery place	Public HCF	1051 (96.1)
	Private HCF	20 (1.8)
	Home	9 (0.8)
	UK / Miss	14 (1.3)
Twin pregnancy	Yes	30 (2.7)
	No	923 (84.4)
	UK / miss	141 (12.9)
Normal delivery	Yes	521 (47.6)
	No	542 (49.5)
	UK / miss	30 (2.7)
Mode of delivery	Normal	780 (71.3)
	Cesarean	277 (25.3)
	UK / miss	37 (3.4)
Cry after birth	Yes	949 (86.7)
	No	124 (11.3)
	UK / miss	21 (2.0)
Resuscitation	Yes	41 (3.7)
	No	1028 (94.0)
	UK / miss	25 (2.3)
Vitamin K	Yes	1034 (94.5)
	No	39 (3.6)
	UK / miss	21 (1.9)

BCG	Yes	516 (47.2)
	No	572 (52.3)
	UK / miss	6 (0.6)
Engerix	Yes	339 (31.0)
	No	736 (67.3)
	UK / miss	19 (1.7)
Gender	Male	606 (55.4)
	Female	488 (44.6)
Birth weight	≤ 1000 g	15 (1.4)
	< 1500 g	41 (3.7)
	< 2500 g	277 (25.3)
	≥ 2500 g	755 (69.0)
	UK / miss	6 (0.6)
Gestational age	< 28 weeks	16 (1.5)
	28 – 31 weeks	59 (5.4)
	32 – 36 weeks	196 (17.9)
	≥ 37 weeks	771 (70.5)
	UK / miss	52 (4.8)

**Characteristics of neonatal period**		**No of cases (%)**
Discharge after birth	Yes	426 (38.9)
	No	654 (59.8)
	UK / miss	14 (1.3)
Admitted from	Home	412 (37.7)
	HCF	152 (13.9)
	OBS	527 (48.2)
	UK / miss	3 (0.3)
Symptoms duration	≤ 1 day	669 (61.2)
	> 1 day	418 (38.2)
	UK / miss	7 (0.6)
Type of feeding	Breast milk	484 (44.2)
	Formula milk	85 (7.8)
	Mix	223 (20.4)
	Not start	285 (26.1)
	UK / miss	17 (1.6)
Difficulties feeding		123 (11.2)
Difficult to wake up		3 (0.3)
Movement only when stimulated		6 (0.5)
Convulsions		9 (0.8)
Difficulties breathing		190 (17.4)
Skin color change	Yellow	297 (27.1)
	Blue	56 (5.1)
	Pale	2 (0.2)
Abnormal stool		9 (0.8)

Characteristics of admission status		No of cases (%)
Ambulance transport	Yes	136 (12.4)
	No	935 (85.5)
	UK / miss	23 (2.1)
Admission ward	PICU	660 (60.3)
	Pediatric Dep.	416 (38.1)
	UK / miss	18 (1.6)
Age at admission	1 day	327 (29.9)
	2 – 7 days	376 (34.4)
	8 – 28 days	369 (33.7)
	UK / miss	22 (2.0)
Skin color	Pink	474 (43.3)
	Yellow	454 (41.5)
	Cyanosis	113 (10.3)
	Pale	45 (4.1)
	UK / miss	8 (0.7)
Temperature	34 – 35.9^{o}C	6 (0.5)
	36 – 38°C	986 (90.1)
	> 38°C	93 (8.5)
	UK / miss	9 (0.8)
Severe respiratory failure	Yes	22 (2.0)
	No	1072 (98.0)
Shock	Yes	26 (2.4)
	No	1068 (97.6)
CNS trouble	Lethargy / coma	59 (5.4)
	No	1035 (94.6)

Characteristics of socio-demographics	Cases (%) N = 1094	Death (%) N = 50	OR (95% Cl)
Ethnic minority			
Kinh	1055 (96,4)	50 (100)	1.0 (NA)
Others	17 (1,6)	0 (0)	-
Mother's education			
≤ Class 5	293 (26,8)	17 (34,0)	1.25 (0.68-2.30)
Class 6 – 12	661 (60,4)	31 (62,0)	1.0 (NA)
> Class 12	115 (10,5)	0 (0)	-
Father's education			
≤ Class 5	167 (15,3)	20 (40,0)	**3.52** (1.90-6.49)
Class 6 – 12	646 (59,0)	25 (50,0)	1.0 (NA)
> Class 12	142 (13,0)	2 (4,0)	0.37 (0.09-1.58)
Number of siblings			
< 2 childs	962 (87,9)	42 (84,0)	1.0 (NA)
≥ 2 childs	92 (8,4)	6 (12,0)	1.53 (0.63-3.70)

Characteristics of pregnancy-delivery	Cases (%) N = 1094	Death (%) N = 50	OR (95% Cl)
ANC visits			
< 3	145 (13.3)	16 (32.0)	**3.50** (1.86-6.57)
≥ 3	906 (82.8)	31 (62.0)	1.0 (NA)
Normal delivery			
Yes	521 (47.6)	8 (16.0)	1.0 (NA)
No	542 (49.5)	42 (84.0)	**5.38** (2.51-11.63)
Multiple pregnancy			
Yes	30 (2.7)	1 (2.0)	0.63 (0.84-4.71)
No	923 (84.4)	48 (96.0)	1.0 (NA)
Delivery place			
Health care facility	1071 (97.9)	47 (94.0)	1.0 (NA)
Home	9 (0.8)	3 (6.0)	**10.89** (2.64-44.91)
Delivery mode			
Normal delivery	780 (71.3)	37 (74.0)	1.0 (NA)
Assisted delivery	25 (2.3)	2 (4.0)	1.75 (0.40-7.69)
Cesarean delivery	277 (25.3)	11 (22.0)	0.83 (0.42-1.65)
Cry after birth			
Yes	949 (86.7)	19 (38.0)	1.0 (NA)
No	124 (11.3)	31 (62.0)	**16.32** (8.87-30.01)
GA			
< 28 weeks	16 (1.5)	6 (12.0)	**65.96** (21.74-200.00)
28 – 31 weeks	59 (5.4)	13 (26.0)	**11.19** (5.21-23.81)
32 – 36 weeks	196 (17.9)	4 (8.0)	0.82 (0.28-2.45)
≥ 37 weeks	771 (70.5)	19 (38.0)	1.0 (NA)
BW			
≤ 1000 gr	15 (1.4)	11 (22.0)	**145.55** (41.67-500.00)
1001 - 1499 gr	41 (3.7)	11 (22.0)	**19.40** (8.13-45.45)
1500 - 2499 gr	277 (25.3)	13 (26.0)	**2.61** (1.21-5.62)
≥ 2500gr	755 (69.0)	14 (28.0)	1.0 (NA)
Gender			
Male	606 (55.4)	29 (58.0)	1.0 (NA)
Female	488 (44.6)	21 (42.0)	0.89 (0.50-1.59)

**Characteristics of neonatal period**	**Cases (%) N = 1094**	**Death (%) N = 50**	**OR (95% Cl)**
Engerix B			
Yes	339 (31.0)	1 (2.0)	1.0 (NA)
No	736 (67.3)	49 (98.0)	**24.11** (3.31-175.33)
BCG			
Yes	516 (47.2)	1 (2.0)	1.0 (NA)
No	572 (52.3)	49 (98.0)	**48.25** (6.64-350.73)
Vitamin K1			
Yes	1034 (94.5)	42 (84.0)	1.0 (NA)
No	39 (3.6)	8 (16.0)	**6.10** (2.64-14.07)
Feeding			
Mother milk	484 (44.2)	9 (18.0)	1.0 (NA)
Formula milk	85 (7.8)	2 (4.0)	1.27 (0.27-5.99)
Mix	223 (20.4)	0 (0)	-
Not started	285 (26.1)	39 (78.0)	**8.37** (3.98-17.54)
Discharge after birth			
Yes	426 (38.9)	3 (6.0)	1.0 (NA)
No	654 (59.8)	47 (94.0)	**10.92** (3.38-35.31)
Symptoms duration			
≤ 1 days	669 (61.2)	49 (98.0)	1.0 (NA)
> 1 day	418 (38.2)	1 (2.0)	**0.03** (0.00-0.22)
Difficult to wake up			
Yes	3 (0.3)	2 (4.0)	**43.46** (3.87-487.67)
No	1091 (99.7)	48 (96.0)	1.0 (NA)
Difficulties eating			
Yes	123 (11.2)	7 (14.0)	1.30 (0.57-2.96)
No	971 (88.8)	43 (86.0)	1.0 (NA)
Movement when stimulated			
Yes	6 (0.5)	1 (2.0)	4.24 (0.49-37.00)
No	1088 (99.5)	49 (98.0)	1.0 (NA)
Convulsions			
Yes	9 (0.8)	1 (2.0)	2.64 (0.32-21.55)
No	1085 (99.2)	49 (98.0)	1.0 (NA)
Difficulties breathing			
Yes	190 (17.4)	32 (64.0)	**9.97** (5.46-18.20)
No	904 (82.6)	18 (36.0)	1.0 (NA)
Skin color changed			
Yes	351 (32.1)	18 (36.0)	1.20 (0.66-2.17)
No	743 (67.9)	32 (64.0)	1.0 (NA)
Abnormal stools			
Yes	9 (0.8)	0 (0)	-
No	1085 (99.2)	50 (100)	1.0 (NA)
Transport time			
Internal	527 (48.2)	34 (68.0)	1.0 (NA)
External	567 (51.8)	16 (32.0)	**0.42** (0.23-0.77)

**Characteristics of admission status**	**Cases (%) N = 1094**	**Death (%) N = 50**	**OR (95% Cl)**
Age at admission			
0 – 1 day	424 (38.8)	45 (90.0)	1.0 (NA)
2 – 6 days	279 (34.6)	4 (8.0)	**0.12** (0.04-0.35)
8 – 28 days	369 (33.7)	1 (2.0)	0.023 (0.003-017)
Skin color			
Pink	474 (43.3)	12 (24.0)	1.0 (NA)
Yellow	454 (41.5)	1 (2.0)	**0.09** (0.01-0.66)
Cyanosis	113 (10.3)	30 (60.0)	**13.92** (6.85-28.29)
Pale	45 (4.1)	6 (12.0)	**5.92** (2.11-16.64)
Temperature			
34 – 35.9^{o}C	6 (0.5)	2 (4.0)	**10.70** (1.91-58.82)
36 – 38°C	986 (90.1)	44 (88.0)	1.0 (NA)
> 38°C	93 (8.5)	4 (8.0)	0.96 (0.34-2.74)
Severe respiratory failure			
Yes	22 (2.0)	10 (20.0)	**21.50** (8.77-52.71)
No	1072 (98.0)	40 (80.0)	1.0 (NA)
Shock			
Yes	26 (2.4)	14 (28.0)	**33.44** (14.44-77.44)
No	1068 (97.6)	36 (72.0)	1.0 (NA)
CNS trouble			
Lethargy or coma	59 (5.4)	23 (46.0)	**23.85** (12.48-45.60)
No	1035 (94.6)	27 (54.0)	1.0 (NA)
